# The PLE^2^NO self-management and exercise program for knee osteoarthritis: Study Protocol for a Randomized Controlled Trial

**DOI:** 10.1186/s12891-016-1115-7

**Published:** 2016-06-07

**Authors:** Priscila Marconcin, Margarida Espanha, Flávia Yázigi, Pedro Campos

**Affiliations:** Universidade de Lisboa, Faculdade de Motricidade Humana, CIPER, LBMF, P-1499-002, Estrada da Costa, 1499-002 Cruz Quebrada, Dafundo, Portugal; CAPES Foundation, Ministry of Education of Brazil, Brasília, DF 70040-020 Brazil; Universidade de Lisboa, Faculdade de Motricidade Humana, CIPER, Laboratório de Fisiologia eBioquímica do Exercício, P-1499-002, Estrada da Costa 1499-002 Cruz Quebrada, Dafundo, Portugal

**Keywords:** Self-management, Exercise, Knee osteoarthritis, Elderly

## Abstract

**Background:**

International recommendations suggest exercise and self-management programs, including non-pharmacological treatments, for knee osteoarthritis (KOA) because they can benefit pain relief and improve function and exercise adherence. The implementation of a combined self-management and exercise program termed PLE^2^NO may be a good method for controlling KOA symptoms because it encourages the development of self-efficacy to manage the pathology. This study will assess the effects of a self-management and exercise program in comparison to an educational intervention (control program) on symptoms, physical fitness, health-related quality of life, self-management behaviors, self-efficacy, physical activity level and coping strategies.

**Methods/Design:**

This PLE^2^NO study is a single-blinded, randomized controlled trial of elderly (aged above 60 yrs old) patients with clinical and radiographic KOA. The patients will be allocated into either an educational group (control) or a self-management and exercise group (experimental). All participants will receive a supplement of chondroitin and glucosamine sulfates. This paper describes the protocol that will be used in the PLE^2^NO program.

**Discussion:**

This program has several strengths. First, it involves a combination of self-management and exercise approaches, is available in close proximity to the patients and occurs over a short period of time. The latter two characteristics are crucial for maintaining participant adherence. Exercise components will be implemented using low-cost resources that permit their widespread application. Moreover, the program will provide guidance regarding the effectiveness of using a self-management and exercise program to control KOA symptoms and improve self-efficacy and health-related quality of life.

**Trial registration:**

NCT02562833 (09/23/2015)

## Background

Osteoarthritis (OA) is the most common type of rheumatic disease [1]. OA is prevalent in elderly populations and has a substantial influence on the health care industry [2, 3]. In the USA, 27 million people, including 12.1 % of the population aged 25–74 years old, are clinically defined as having OA [4].

OA is an active disease [5] that affects all articular tissues [6]. OA can be characterized by examining a person’s symptoms, especially pain [7], which influence the performance of daily living activities [8] and psychological parameters [3]. Among older adults, OA primarily affects weight bearing joints, such as the knee and hip, and is therefore a cause of lower extremity disability [9]. In Portugal, knee OA (KOA) is considered to be the third most prevalent rheumatic disease (affecting 12.4 % of the population) [10].

Most types of interventions that are aimed at managing KOA involve community and primary care [5]. Hence, it is imperative to consider international recommendations that can assist individuals and that are feasible alternatives to health services. The Osteoarthritis Research Society International (OARSI) [11], the American College of Rheumatology (ACR) [12] and the European League Against Rheumatism (EULAR) [13] strongly recommend exercise (including land-based, such as strengthening and aerobic activity or water-based activities) and self-management programs as non-pharmacological treatments for KOA patients.

### Self-management programs

Patient education, information and self-management support are critical for patient cooperation during treatment. Besides OARSI international recommendations (11), several evidence-based studies of self-management programs have demonstrated that it is effective to empower patients to better manage their own chronic diseases [14–26].

Psychoeducational interventions are growing in popularity in the primary care field [24]. Among these efforts, self-management programs deserve special attention. The following three models of chronic disease self-management programs are the most widely used: the Expert Patient Programme [27], the Flinders Model [28], and the Stanford Model [29]. The Expert Patient Programme focuses on increasing patient knowledge to manage conditions, the Flinders Model emphasizes the role that physicians play in building patient self-efficacy, and the Stanford Model uses peer educators to build self-efficacy [30].

Two programs have followed the format of the Stanford Model. These include the Arthritis Self-Management Program (ASMP) and the Chronic Disease Self-Management Program (CDSMP) [31, 32]. The first of these, the ASMP, is a specific program for people with arthritis that was developed in the 1970s at the Stanford Patient Education Research Center [19]. Later, the same group developed a more generic proposal for patients with any chronic condition, the CDSMP. This program has now spread in popularity throughout the US [31, 32] and other countries [17, 18, 23, 25, 33].

A meta-analysis of the ASMP and the CDSMP [34] revealed that improvements were observed in several outcome measures in patients with chronic diseases at 4 and 12-month follow-ups.

### Exercise programs

Studies have demonstrated that exercise benefits patients with KOA [35–46]. The two most recognized approaches for KOA treatment with exercise are land-based [47–49] and aquatic programs [44, 50–53]. A recent systematic review and meta-analysis [54] provided evidence showing that land-based exercise is beneficial for people with KOA because it reduced joint pain and improved physical function and quality of life over the short-term and for at least two to six months after the cessation of treatment. Regarding the exercise mode, studies have demonstrated that there is no difference between the efficacies of strengthening, flexibility plus strengthening, flexibility plus strengthening plus aerobic exercise, aquatic strengthening, aquatic strengthening plus flexibility and a combined intervention that included strengthening, flexibility, and aerobic exercise when each was compared to a no exercise control, and there were no differences between the effect of the interventions on improving functional limitations in people with lower limb OA [55]. Additionally, no difference was observed in the effectiveness of providing pain relief between strengthening and aerobic exercises across eight studies that involved KOA patients [56].

Thus, combining aerobic and muscle strengthening exercises into a single program may produce even better outcomes in arthritis patients [57] than programs based on only one of these components. A program that combined aerobic and resistance exercises significantly improved physical function and daily living activities and reduced pain in older adults with arthritis [40], as well as decreased depression [36, 49, 58, 59]. Another program combined a variety of exercises focused on core strength and balance, flexibility, upper and lower body strength and aerobic conditioning and resulted in improvements in mobility, aerobic endurance, strength, flexibility, and self-reported pain perception [35].

### Nutritional supplements: glucosamine and chondroitin sulfate

Although important, controlling symptoms is not the only target when treating OA patients. Indeed, an ideal treatment for OA should preserve joint structures, improve quality of life and for drug therapy or supplementation, have a good safety profile [60]. It is paramount that the administrator account for side effects that can result from the chronic use of OA pharmacological therapies, such as nonsteroidal anti-inflammatory drugs (NSAIDs) [61]. Therefore, glycosaminoglycans such as chondroitin sulfate (CS) and glucosamine sulfate (GlcN-S) are two natural supplements that are considered to be symptomatic slow-acting drugs for osteoarthritis (SYSADOA) [60].

GlcN-S has been shown to exhibit structure-modifying effects, including small to moderate protective effects on minimum joint spaces after 3 years, in KOA patients [62]. This finding was in agreement with the results of a previous trial that indicated that GlcN-S prevents total knee replacement (TKR) [63].

CS has also been evaluated in different clinical trials that have sought to document both its symptomatic potential and its structure-modifying effects. A recent study [64] demonstrated the efficacy of CS for treating symptoms (i.e., pain and lower-limb function) and concluded that CS is an efficient and safe intervention. Interestingly, CS produced a significant reduction in joint swelling and effusion in a gait study [65].

A double-randomized placebo-controlled clinical trial with a 2-year follow-up of 605 patients with KOA demonstrated that after adjusting for factors associated with structural disease progression, a dietary supplement that consisted of a combination of GlcN-S and CS resulted in significantly less joint space narrowing than was observed with the placebo, whereas neither CS nor GlcN-S alone was effective [66]. A combination of GlcN-S-hyaluronic acid (500 mg) and CS (400 mg) was found to be efficient at providing pain relief and functional improvement in OA patients with moderate to severe knee pain [65]. These findings suggested that a combination of GlcN-S and CS may be more efficient than either CS or GlcN-S alone.

Although some interventions have combined patient self-management with an exercise component, we were unable to identify any study that combined these components with GlcN-S and CS supplementation.

### Aim and hypothesis

The aim of this study is to design and implement a PLE^2^NO program (in Portuguese: Free Program of Education and Exercise for Osteoarthritis) for elderly patients with KOA for a duration of three months. The PLE^2^NO is based on applying the principle of self-efficacy to manage the pathology. When patients gain confidence in taking control of their disease, they are more comfortable exercising and managing OA symptoms and consequently make better decisions about treatment. This allows them to increase their quality of life. To encourage participant’s adherence to and maintenance of the program and to contribute to pain control, all participants will receive a supplement containing CS and GlcN-S.

The following three hypotheses were therefore formulated. H1, self-reported KOA symptoms (i.e., pain and stiffness) and physical fitness will improve more in the self-management and exercise group than in the control group; H2, self-management skills and self-efficacy will improve more in the self-management and exercise group than in the control group; and H3, health-related quality of life, physical activity levels and coping strategies will improve more in the self-management and exercise group.

## Methods and design

### Study design

**Fig. 1:**
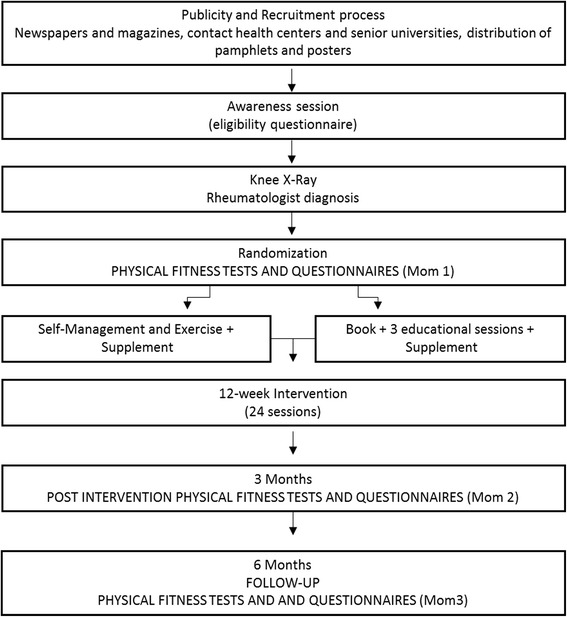
The PLE^2^NO flowchart

The PLE^2^NO is a single-blinded, randomized controlled trial with a three-month duration and a six-month follow-up. The participants will be individually randomly assigned to one of two groups: (1) a self-management and exercise group or (2) an educational control group. Both groups will receive supplementation (CS and GlcN-S). Figure 1 provides a flowchart of the PLE^2^NO design. It will not be possible to blind the participants because of the nature of the intervention. However, the assessors will be blinded to group allocation.

### Sample size

The sample size was calculated based on the primary outcome (self-reported pain). The analysis were done by the program GPower 3.1 [67], based on a priori analysis with ANCOVA, using one covariate and two groups with 80 % power at a 5 % significance. According to McKnight’s study [68], a combined strength training and a self-management program, we fixed the effect size on 0.35 and determined we needed a total sample size of 67. Considering a possible dropout of 20 %, we aimed to recruit 80 subjects and allocated 40 subjects per group.

### Participants and procedures

The recruitment and selection processes will be performed using the following eligibility criteria: (1) an age of 60 years old or older, (2) bilateral or unilateral KOA diagnosed according to the clinical and radiological criteria of the American College of Rheumatology (ACR) [69], and (3) participants who are independently mobile and literate. The exclusion criteria will be the following: (1) involvement in another intervention program (exercise, education or physical therapy), (2) the prior use of supplements (chondroitin and/or glucosamine sulfate) for at least three months, and (3) other pathologies (e.g., cardiovascular, respiratory, and musculoskeletal pathologies and cancer) that prevent the practice of physical exercise, (4) a mental/psychological state that hinders understanding the program, (5) surgery for knee replacement or a plan to undergo surgery to place a prosthesis within the next eight months, (6) an allergy to shellfish or another component of the supplements, and (7) administration (injections) of corticosteroids or hyaluronic acid in the last 6 months.

To avoid convenience sampling, the participants will be recruited from the Lisbon area, and different marketing strategies will be used to advertise and publicize the PLE^2^NO program. Social networks, newspapers, magazines, contacts with senior universities, health centers, churches and community centers, and the site of the Portuguese League Against Rheumatic Disease will be the main channels used for PLE^2^NO announcements.

All individuals interested in participating will be invited to an awareness session in which the details of the program will be explained, and the patients will complete an eligibility questionnaire, which is necessary to acquire more detailed information, including whether they have any allergies to components in the supplements. As many sessions as necessary will be performed until the expected sample size is attained. Anyone who is interested and fulfills the eligibility criteria will receive a request for an x-ray examination. The exam requests will be referred to a rheumatologist who will make the final diagnosis according to the ACR clinical and radiological criteria. This is a more specific diagnosis (86 %) than a simple clinical diagnosis (69 %) [70]. If the subject is found to be positive for KOA, he or she will be invited to a second interview during which consent will be obtained.

The randomization process will be performed on the baseline assessment day by the research team leader. The randomization sequence will be a 1:1 allocation to the two treatment arms.

### Interventions

The active treatment group will engage in self-management and exercise (SMEG), and the control group will engage in patient education (EG) only. The SMEG patients will receive a combination program including self-management and exercise that will be delivered on the same days twice per week. Each session will last 90 min. The first 30 min will be allocated for self-management, and the remaining 60 min will be used for exercise. The program will be offered in a group format that encourages interaction and socialization, which can help to counteract feelings of depression and isolation. To avoid any conflict of interest and because we believe that it will help support the participants’ adherence, maintenance and pain control, all participants will receive a supplement that consists of a combination of two main substances: 1500 mg of glucosamine sulfate and 1200 mg of chondroitin sulfate, in addition to two secondary substances: 100 mg of *Harpagophytum* extract and 10 mg of hyaluronic acid. The recommendation is to use two packets per day. The participants themselves will have to complete daily sheets that request information regarding pain levels that are assessed on a visual numeric pain scale [71] and a bi-daily supplementation diary. All participants will be covered by personal accident insurance.

### Self-Management and Exercise Group (SMEG)

#### Self-management component

The self-management component is based on a program that was developed at Stanford University, the Chronic Disease Self-Management Program (CDSMP) [32], which aims to develop self-efficacy and emphasizes skills mastery. These are accomplished through the weekly performance of specific behaviors and the receipt of feedback (action plan and problem solving). The contents of the program will include the following: self-management principles, managing symptoms, exercise and physical activity, communication skills, healthy eating, and managing medicines. The program will be administered by a certified Master Trainer and Leader of the CDSMP at Stanford University.

#### Exercise component

The exercise component is based on the Fit and Strong Program [72], Exercise for People with Arthritis (FEPA) [35] and the Taking Control with Exercise (Arthritis Foundation) program. This exercise program contains health-related (muscular resistance/strength, and flexibility) and skill-related (balance) physical fitness components. Additionally, the program will include socialization games that help to decrease symptoms related to pain, stress, depression, and fatigue. In addition to improvements in physical fitness, the development of self-efficacy in exercise is another goal.

The exercise session type includes a warm up for the first 5 min, followed by 15 to 20 min of recreation activity and balance exercise, 30 to 40 min of the strengthening exercises, and 10 to 15 min of stretching and relaxation exercises at the end.

Specific strength exercises will be performed to recruit specific muscle groups in the lower limbs (quadriceps, hamstrings, hip adductors/abductors, gluteus, and gastrocnemius) and the upper limbs (pectoralis, trapezius, dorsal, deltoids, biceps and triceps). The strength exercises will use a combination of elastic bands (upper limbs) and cuff weights (lower limbs) or calisthenics, as previously used in other studies [73, 74] and replicated in the Fit and Strong program [72]. The resistance will be progressively increased throughout the program by adding weights in increments of 0.250 Kg to the cuff weights. The progressions in the numbers of repetitions and series are illustrated in Table 1.

**Table 1:** Training volume

Week 1-3	Week 4-6	Week 7-9	Week 10-12
No additional load	Load 1	Load 2	Load 3
Week 1: 1 × 12 rep	Week 4: 1 × 12 rep	Week 7: 1 × 12 rep	Week 10: 1 × 12 rep
Week 2: 2 × 8 rep	Week 5: 2 × 8 rep	Week 8: 2 × 8 rep	Week 11: 2 × 8 rep
Week 3: 2 × 12 rep	Week 6: 2 × 12 rep	Week 9: 2 × 12 rep	Week 12: ×12 rep

The prescribed intensity and management of exercise resistance will be primarily guided by answers related to self-reported pain, which will be assessed using a Visual Numeric Pain Scale [71] before, during and after each session. At the beginning of each session, all participants will be required to present their pain diaries. If the pain level is above five points on the day before the last session, the load will not be increased, but if pain is below five points, they patients will receive increased loads. The intensity interval desired for strengthening exercises will be maintained at 4-6 (somewhat easy – somewhat hard) according the Omni-Perceived Exertion Scale for Resistance Exercise (OMINI-RES) [75].

This component will be overseen by a professional with a master’s degree in Science of Physiotherapy and another individual who is an Exercise and Health master’s student. Both will be from the Faculty of Human Kinetics.

To develop exercise self-efficacy and promote the maintenance of the exercise program before the end of the class, a chart with the main exercises for each physical fitness component (i.e., muscular resistance/strength, flexibility, and balance) will be given to the participants during the last two weeks of the intervention program. The participants will be encouraged to perform the exercises by themselves by following the chart during the last two weeks with supervision from the same professionals that conducted the exercise program. It is expected that they will develop the capacity to perform the exercises by themselves in their homes without supervision by the end of the intervention.

### Education control Group (EG)

This group will receive a book [76] published by PLE^2^NO’s scientific team. This book contains descriptions and tips for managing KOA in addition to educational and exercise information presented as images. Additionally, the participants will attend three monthly educational sessions that are one hour in length each regarding joint protection strategies, exercise, and self-management techniques. These sessions will be delivered by the coordinator of the PLE^2^NO project, who is a PhD Professor in the Faculty of Human Kinetics, and an Exercise and Health master’s student from the same institution. Telephone contacts will be established 15 days after each educational session to avoid withdrawals and to maintain closer monitoring.

### Assessments and procedures

The assessments will be performed one week prior to the start of the program (baseline), during the week following the final intervention (three months later), and at a 6-month follow-up, and will be done by PLE^2^NO team member(s) (all of whom are master students in exercise and health specialties) who are blinded to group allocation. Each evaluator performs the same test to avoid inter-rater variability. The assessments will be performed on the same day. To avoid overloading the participants, the physical tests and questionnaires will be performed alternately. Additionally, the physical tests involving load-bearing activities will be alternated with those that are performed while seated. The order of those tests and questionnaires were determined previously, in accordance with the statements already mentioned.

The main outcome will be self-reported pain (sub-score of the Knee Injury and Osteoarthritis Outcome Score –KOOS). The secondary outcomes will be: other KOA symptoms, KOA-specific health-related quality of life, self-efficacy, self-management behaviors, a healthier quality of life, a physically active lifestyle, coping strategies, aerobic capacity, functional strength, mobility, flexibility, gait speed, static balance and handgrip. All outcomes and instruments are illustrated in Table 2 and will be assessed at baseline, post-intervention and a 6-month follow-up.

**Table 2:** Outcomes and instruments

	Outcomes	Instruments
Questionnaires	KOA-specific health-related quality of life	Knee Injury and Osteoarthritis Outcome Score (KOOS)
	Self-efficacy	Self-efficacy for managing Chronic Disease 6-Item Scale
	Self-management behaviors	Cognitive Symptom Management and Communication with Physicians
	Health-related quality of life	Euroquol five dimensions five level (EuroQol -EQ-5D-5L)
	Physical activity	International Physical Activity Questionnaire (IPAQ)
	Coping strategies	Brief COPE
Physical fitness tests	Aerobic Capacity	Six-Minute Walking Test (6 MWT)
	Functional lower-limb strength	Five-Repetition Sit to Stand Test (FRSTST)
	Mobility	Timed “Up-and-Go” test
	Flexibility upper limb	Back Scratch Test (BST)
	Flexibility lower limb	Chair Sit and Reach (CRS)
	Gait speed	6-Meter Test
	Balance	Standing Balance
	Hand strength	Hand grip test

#### Eligibility questionnaire

This questionnaire collects personal data (including name, phone contact, address, and email) and the inclusion and exclusion criteria for participation in the program. It will be available both online and on paper.

#### X-ray

Bilateral, anterior-posterior knee radiographs will be used to identify OA in the tibiofemoral joint, and sunrise views will be used to identify OA in the patellofemoral compartment. The severity of OA in the tibiofemoral and patellofemoral joint will be measured by a rheumatologist using the K-L grading scale [77].

#### Socio-demographic information

A questionnaire will be created by the researchers that poses demographic questions, including date of birth, race, sex, marital status, current occupation, occupation before retiring and education level. Body mass index (BMI) will also be calculated as weight (measured in kilograms) over height squared (height measured in meters).

#### Use of medicine

A list containing the names of all medications being used and their doses and indications will be requested from the patients before and after the intervention (baseline and post-intervention).

### Questionnaires

#### Knee Injury and Osteoarthritis Outcome Score (KOOS)

This questionnaire includes 5 dimensions to measure KOA-specific health-related quality of life (QOL), knee pain (Pain), other disease-specific symptoms (Other Symptoms), daily living activities (ADL), and sport/recreation functions (Sport/Rec). A score for each of the five dimensions is calculated as the sum of the items that are included, which is then converted to a 0–100 scale in which 0 represents extreme knee problems and 100 represents no knee problems. The KOOS has been validated for use in patients with knee injuries and patients with KOA and is a reliable and responsive self-administered instrument for short-term follow-ups [78].

#### Self-efficacy for managing chronic disease 6-item scale

This 6-item scale contains items taken from several self-efficacy scales that were developed for the Chronic Disease Self-Management study. This is a one to ten scale that includes six questions. The scale was tested on 605 subjects with chronic diseases [31]. The observed range of outcomes was 1-10 with a mean of 5.17, a standard deviation of 2.22, and an internal consistency reliability of 0.9.

#### Cognitive symptom management

This scale comprises six questions and has an observed range of 0–5. The scale was tested on 1129 subjects with chronic disease, and 51 of these subjects who underwent a test-retest protocol [79]. The mean result was 1.33 with a standard deviation of 0.91, an internal consistency reliability of 0.75 and a test-retest reliability of 0.83.

#### Communication with physicians

This questionnaire includes three questions. The scale was tested on 1130 subjects with chronic disease, and 51 of these subjects underwent a test-retest protocol [79]. The results showed an observed range of 0–5, a mean of 3.08, a standard deviation of 1.20, an internal consistency reliability of 0.73 and a test-retest reliability of 0.89.

#### Perception of health and quality of life (EuroQol - EQ-5D-5 L)

The EQ-5D-5L is a generic instrument for measuring health-related quality of life (HRQoL) that allows the generation of an index that represents a status value of the health of an individual. This scale is based on a classification system that describes health along the following five dimensions: mobility, personal care, usual activities, pain/discomfort, and anxiety/depression. Each of these dimensions has five levels of severity. This instrument employs psychometric techniques similar to those of the EQ-5D [80].

#### International Physical Activity Questionnaire (IPAQ)

The short form of the IPAQ was chosen because it is easy to apply. Its reliability has been verified in many countries and in different populations [81, 82].

#### Brief COPE

The first version of the COPE inventory by Carver, Scheier and Weintraub [83] was subsequently abbreviated by Carver [84]. The abridged version (brief COPE) contains only 28 items that are answered on a Likert 4-point scale (ranging from 1 = never use this strategy to 4 = I often use this strategy) and divided into the following 14 sub-scales (two items per scale): active coping, denial, substance use, emotional support, instrumental support, behavioral divestment, ventilation, revaluation, planning, mood, acceptance, religion, and self-blame. Data from a study of survivors of Hurricane Andrew indicate that the brief COPE scales have an adequate internal reliability [84].

### Physical fitness tests

#### Six-Minute Walk Test (6 MWT)

This test is a valid measure of aerobic capacity in older adults [85], and it has been used in studies of KOA [86, 87].

#### Five-Repetition Sit to Stand Test (FRSTST)

This measure is a widely used measure of functional strength. The ICC values for this test reveal good to high test-retest reliability for adults and subjects with osteoarthritis [86, 88, 89].

#### Timed “up-and-go”

This is a test of strength, agility and dynamic balance that incorporates multiple activity themes. The time (seconds) taken to rise from a chair, walk 3 m (9 ft, 10 inches), turn, walk back to the chair and then sit down wearing regular footwear (while using a walking aid if required) is assessed [90].

#### Chair Sit and Reach test (CSR)

The CSR test is a safe and socially acceptable alternative to traditional floor sit-and-reach tests and is a reasonably accurate and stable measure of hamstring flexibility [91]. The subjects are allowed three attempts for each limb, and the best of these scores is recorded to the nearest centimeter.

#### The Back Scratch Test (BST)

The BST is a measure of overall shoulder range of motion. This test involves measuring the distance, using a ruler, between (or overlap in) the middle fingers when they are placed behind the back [92]. After a practice trial, this test is assessed twice, alternating between both hands, and the best value is registered for each.

#### Six-meter test

This test measures linear walking ability, excluding acceleration and deceleration [93]. This variable is also used as a primary outcome in an algorithm for sarcopenia in older individuals [94].

#### Standing balance test

This test will be performed bilaterally. While near a wall, the subject crosses theirs arms over their chest, lifts the preferred leg off the floor without touching the other leg, and holds this position with their eyes open as long as possible. Contact between the legs, the support touching the ground, touching the wall and withdrawing the arms from the chest are considered errors. The evaluator stops recording the time upon the occurrence of any error. The participants will perform two repetitions of the test, and the best result will be recorded [95].

#### Hand Grip Test (HGT)

This test evaluates the maximal isometric force exerted by the muscles of the hand and forearm using a dynamometer. Although this study will not examine hand OA, this test has been used in the elderly as an indicator of sarcopenia and/or disability [96, 97]. Prior to the test, the grip dynamometer will be adjusted to the size of the hands of each subject. The subjects will stand with their arms along their bodies without contact with their trunk and with their elbows slightly bent at a 20° angle. Testing will first be conducted using the dominant hand and subsequently using the non-dominant hand. Strength will be evaluated during the expiratory phase to avoid the Valsalva maneuver. The best of three repetitions will be chosen for further analysis.

### Other measures

#### Patient’s Global Impression of Change (PGIC)

This scale is often used in clinical research, particularly in musculoskeletal studies [98]. The changes will be classified on dichotomous scales, and the classifications that will be used will include perceived change (5–7), an experience reflecting significant changes (1–4) and a lack of experience reflecting significant changes [98].

#### Visual Numeric Pain Scale (VNS)

This scale is used to self-report pain. It combines strong visual cues with an 11-point numeric rating scale. The VNS is highly correlated with the visual analogue scale (VAS, r = 0.85), is sensitive to changes in pain, and has been demonstrated to be a valid measure [71].

#### OMNI resistance exercise scale

This scale is a perceived exertion scale used with resistance exercise, and its high level of construct validity indicates that the OMNI-RES measures the same properties related to exertion as the Borg RPE scale [99] during resistance exercise [75].

### Data analysis

The data will be analyzed in a blinded manner. Descriptive statistics will be used to describe subject characteristics. The intervention and control groups will be examined for baseline comparability with respect to demographic and other factors. Kolmogorov-Smirnov tests will be used to test for normality. Univariate analyses of covariance (ANCOVA) will be conducted to compare changes between groups (i.e., the self-management and exercise group compared to the educational group) with adjustments for baseline values. The mean difference within groups will be calculated as Mom 1 (baseline) minus Mom 2 (after intervention program). The effect sizes will be verified using partial eta squared statistics. Repeated measures analyses using linear mixed models will be used to assess the constancy of any effects in the self-management and exercise group over time. Missing data will be assumed to be missing at random. All statistical analyses will be performed using IBM SPSS Statistics 22.0 and MedCalc Statistical Software (MedCalc Software, Mariakerke, Belgium), and significance will be established at a level of 5 %.

## Discussion

It is essential to identify the best approach to treating patients with KOA. Such an approach should consider the individuals’ quality of life, international recommendations for treatment and the availability of health services. Therefore, the combined use of self-management, exercise and supplements (glucosamine and chondroitin sulfate) appears to be a feasible and effective option for treating elderly patients with KOA.

There are several strengths to the design and implementation of this study protocol. First and foremost, this program combines the recommendations of international organizations (OARSI, EULAR and ACR) with a combination of exercise and educational (self-management) programs. The study design is extremely current, ambitious and grounded.

Second, the program will be administered in close proximity to the patients. To achieve this goal, the program will take place at four different locations: two senior universities, one church, and one community center. This is necessary because when we consider the age and pathological conditions that we expect to find in the study patients, locomotion may be a barrier. Therefore, if a patient will not be able attend for financial reasons, a van from a church or a team member’s car will provide transportation services. These efforts will minimize the problem of access to the classes.

Third, the methodology of the program, in terms of both self-management and exercise, has been planned in extreme detail using simple resources, including paper roles for the self-management component and elastic bands, ankle weights and chairs for the exercise components. Thus, the program can be feasibly disseminated (e.g., it uses minimal, low-cost equipment and has few storage requirements). The exercise program will be administered by highly qualified exercise instructors, two of whom have master’s degrees in Sport Science and the Science of Physiotherapy, and one other instructor who is an Exercise and Health master’s student from the Faculty of Human Kinetics. All of these instructors specialize in exercise, health and fitness group skills. Furthermore, a certified leader of the Chronic Disease Self-Management Program (CDSMP) at Stanford University will administer the self-management program.

The program will also have a self-efficacy component for exercise, with a goal that following the end of the program, the patients will continue doing exercises, and they will receive support in this endeavor, including access to the materials that were used in the program, a chart with a description of all of the exercises that they performed in class and a brief explanation about how these exercises should be performed.

With the exception of the knee radiographs for the OA diagnoses, all measurements will be obtained at the same place at baseline immediately after the end of the program and at the 6-month follow-up. Therefore, to support the project, the staff team includes one secretary who is responsible for the administrative work and four health professionals who will conduct the tests and questionnaires. To avoid inter-rater error, the same health professionals will lead the applications of the three assessments, i.e., the baseline, post-intervention and follow-up assessments.

Participant adherence to the exercise program is one of the main challenges, mainly because the participants are elderly and susceptible to other health problems. Therefore, motivational cues, intragroup social interactions, frequent telephone calls and the quality of the professors are the main strategies that have been selected to prevent the occurrence of dropouts.

One possible constraint to the success of the program is the extensive exclusion criteria, but these criteria are required to maintain the quality of the study. In this study, all adverse events will be documented and reported from screening until study completion.

Our study is based on the premise that elderly patients with KOA need an appropriate treatment regimen that is accessible and achievable, given their condition. Therefore, the study treatment regimen was designed to develop their self-efficacy to manage their own condition. The concepts of autonomy, self-management and self-efficacy are therefore essential. Moreover, once the program ends, the participants are expected to continue the treatment using self-management skills and by performing the exercises on their own, which should consequently assist them in coping better with pain and KOA symptoms.

The findings of this study will contribute to clinical trial reference data for elderly individuals with KOA by adding information regarding the effectiveness of combining a self-management strategy with an exercise program.

The format of the sessions, the study duration and the weekly frequency of the program are organized in a manner that ensures that this proposal is executable not only for this project but also for future implementations by communities.

## Conclusion

This study is a randomized controlled trial (RCT) that uses a self-management and exercise intervention strategy along with glucosamine and chondroitin sulfate supplementation. The protocol was specially designed according to a carefully controlled methodology. The projected results will enable the implementation of a new combination treatment for elderly patients with KOA.

## Abbreviations

6 MWT, six-minute walking test; ACR, American College of Rheumatology; ASMP, The arthritis self-management program; BMI, body mass index; BST, back scratch test; CDSMP, chronic disease self-management program; CS, chondroitin sulfate; CSR, chair site and reach; EG, education control group; EQ-5D-5 L, EuroQuol five-dimension five-level; EULAR, European League Against Rheumatism; FRSTST, five repetitions sit to stand test; GlcN-S, glucosamine sulfate; IPAQ, international physical activity questionnaire; KOA, knee osteoarthritis; KOOS, knee injury and osteoarthritis outcome score; NSAIDs, nonsteroidal anti-inflammatory drugs; OA, osteoarthritis; OARSI, Osteoarthritis Research Society International; PGIC, patient’s global impression of change; PLE^2^NO, (in Portuguese: Free Program of Education and Exercise for Osteoarthritis); SMEG, self-management and exercise group; SYSADOA, symptomatic slow-acting drugs for osteoarthritis; TKR, total knee replacement
